# Smart Photovoltaic Windows for Next‐Generation Energy‐Saving Buildings

**DOI:** 10.1002/advs.202407177

**Published:** 2024-10-01

**Authors:** Qian Wang, Zongxu Na, Li Yu, Songyuan Dai, Mohammad Khaja Nazeeruddin, Huai Yang

**Affiliations:** ^1^ Institute for Advanced Materials and Technology University of Science and Technology Beijing Beijing 100083 China; ^2^ Hubei Key Laboratory of Plasma Chemistry and Advanced Materials School of Materials Science and Engineering Key Laboratory of Green Chemical Engineering Process of Ministry of Education Wuhan Institute of Technology No. 206 Guanggu 1st Road Wuhan 430205 China; ^3^ State Key Laboratory of Alternate Electrical Power System with Renewable Energy Sources North China Electric Power University (NCEPU) Beijing 102206 China; ^4^ Institute of Chemical Sciences and Engineering, École Polytechnique Fédérale de Lausanne (EPFL) Lausanne CH‐1015 Switzerland; ^5^ School of Materials Science and Engineering Peking University Beijing 100871 China

**Keywords:** chromic materials, energy‐saving buildings (ESBs), light adjusting, photovoltaic performance, smart photovoltaic windows (SPWs), solar cells

## Abstract

The global energy system transforming from fossil fuels to renewable green energy through the adaption of innovative and dynamic green technologies. Energy‐saving buildings (ESBs) are attracting extensive attention as intelligent architectures capable of significantly reducing the energy consumption for heating, air‐conditioning, and lighting. They provide comfortable working and living environment by regulating and harnessing solar energy. Smart photovoltaic windows (SPWs) offer a promising platform for designing ESBs due to their unique feature. They can modulate solar energy based on dynamic color switching behavior under external stimuli and generate electrical power by harvesting solar energy. In this review, the‐state‐of‐art of strategies and technologies are summarized putting SPWs toward high‐efficiency ESBs. The SPWs are systematically categorized according to the working principle and functional component. For each type of SPWs, material and architecture engineering are focused on to optimize operation mode, optical modulation capability, photovoltaic performance and durability for giving ESBs flexible manipulation, extraordinary energy‐saving effect, and high electricity power. In addition, the challenges and opportunities in this cutting‐edge research area are discussed, with the aim of promoting the development of advanced multifunctional SPWs and their application in high efficiency ESBs.

## Graphical abstract

Smart photovoltaic windows (SPWs) are intelligent devices possessing optical modulation and photoelectric conversion characteristics, consequently offer a promising platform for exploiting energy‐saving buildings (ESBs). This article reviews the‐state‐of‐art of strategies and technologies for exploring advanced functional SPWs with flexible manipulation, extraordinary energy‐saving effect, and high electricity power which enables them for next‐generation ESBs.

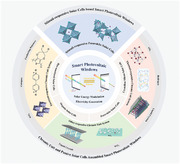

## 1 Introduction

Escalating energy and environmental crises propel researchers across academic and industrial fields to explore green technologies for effective and sustainable energy utilization.^[^ ^1^ ^]^ As the energy use in buildings encompassing indoor heating, air‐conditioning, lighting and ventilation accounts for ≈40% of global energy consumption, the construction of energy‐saving buildings (ESBs), an intelligent system that can improve resource utilization and building efficiency to minimize the energy consumption via regulating and harnessing solar energy, has become a hot topic in recent years.^[^ ^2^, ^3^ ^]^

**Figure 1:**
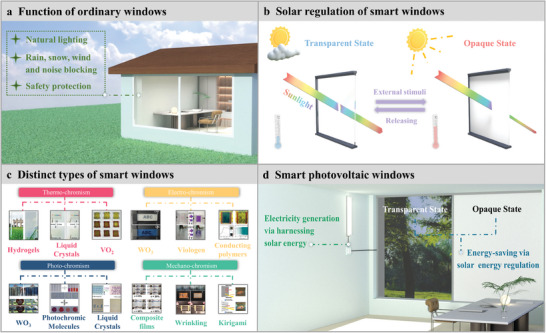
a) Functions of ordinary windows in the interaction between the occupant and external environment. b) Schematic of smart windows regulating solar energy according to the weather condition to give energy‐saving effect. c) Distinct types of smart windows. Reproduced with Permission.^[^ ^6^ ^]^ Copyright 2023, Wiley‐VCH. Reproduced with Permission.^[^ ^7^ ^]^ Copyright 2017, Royal Society of Chemistry. Reproduced with Permission.^[^ ^8^ ^]^ Copyright 2024, Elsevier. Reproduced with Permission.^[^ ^9^ ^]^ Copyright 2018, American Chemical Society. Reproduced with Permission.^[^ ^10^ ^]^ Copyright 2015, Wiley‐VCH. Reproduced with Permission.^[^ ^11^ ^]^ Copyright 2022, Royal Society of Chemistry. Reproduced with Permission.^[^ ^12^ ^]^ Copyright 2015, Wiley‐VCH. Reproduced with Permission.^[^ ^13^ ^]^ Copyright 2016, Optica Publishing Group. Reproduced with Permission.^[^ ^14^ ^]^ Copyright 2019, Elsevier. Reproduced with Permission.^[^ ^15^ ^]^ Copyright 2024, Wiley‐VCH. Reproduced with Permission.^[^ ^16^ ^]^ Copyright 2014, Elsevier. Reproduced with Permission.^[^ ^17^ ^]^ Copyright 2019, American Chemical Society. d) Key features of SPWs.

Windows, a key building component, play an important role in the interaction between the occupant and the external environment.^[^ ^4^ ^]^ They provide the occupant with a clear view to enjoy outside scenery, which is beneficial from the transparency. They can also block the wind, rain, snow, and noise to give the occupant a comfortable, safe, independent working and living space (**Figure** **1**a). By integrating chromic materials and structures into the designing of windows, the windows become an intelligent framework, namely smart windows, that achieve solar energy modulation capability via dynamically changing their transparency under external stimuli.^[^ ^5^ ^]^ Due to this unique feature of smart windows, they provide energy‐saving effect by regulating the propagation of solar energy on demand according to the weather conditions (Figure 1b). On the one hand, smart windows are kept in an opaque or colored state on hot days to block the entrance of the sunlight indoors, reduce the thermal‐effect of the sunlight and consequently decrease the energy consumption for air‐conditioning. On the other hand, smart windows are switched to a transparent or bleached state on cold days to effectively utilize the thermal‐effect of the sunlight to reduce the energy consumption of the heating. To obtain outstanding energy‐saving effect, a variety of thermal‐,^[^ ^6^, ^7^, ^8^ ^]^ electrical‐,^[^ ^9^, ^10^, ^11^ ^]^ mechano‐,^[^ ^12^, ^13^, ^14^ ^]^ and photo‐responsive^[^ ^15^, ^16^, ^17^ ^]^ smart windows with distinct compositions and architectures have been devised (Figure 1c). Besides the energy‐saving effect, solar energy harvesting and utilization characteristics are indispensable for high‐efficiency ESBs.

Consequently, a new concept, “smart photovoltaic windows” (SPWs) is proposed.^[^ ^18^ ^]^ SPWs are intelligent devices combining energy‐saving and electrical power output by regulating and harnessing solar energy (Figure 1d). SPWs have been considered an ideal candidate for exploiting high efficiency ESBs due to their significant features. Similar to smart windows, they can offer energy‐saving effect because of their dynamical transparency‐switching behaviors under external stimuli.^[^ ^19^, ^20^ ^]^ In addition, they can capture solar energy and directly convert it into electrical output which cannot be achieved in smart windows.^[^ ^21^, ^22^ ^]^ The electrical output generated by SPWs via utilizing solar energy can be potentially applied for driving their dynamical transparency‐switching behaviors and compensating the routine energy consumption of ESBs.^[^ ^23^, ^24^ ^]^ Despite several reviews that have been published in this burgeoning field, they mainly focus on SPWs deriving from perovskite solar cells or electric chromic materials.^[^ ^25^, ^26^, ^27^, ^28^, ^29^, ^30^ ^]^ In this feature article, we comprehensively summarize the latest activities of SPWs. Initially, the developed SPWs are categorized into stimuli‐responsive solar cells based SPWs and chromogenic unit and passive solar cells assembled SPWs according to their work principle. Then, material and architecture engineering to achieve critical performance, such as the operation mode, energy‐saving effect, power conversion efficiency (PCE), and stability required for building up high efficiency ESBs, are discussed in each type of SPW. Finally, a few remarks are made in terms of the opportunities and challenges for exploring advanced multifunctional SPWs toward high efficiency ESBs.

## 2 Stimuli‐Responsive Solar Cells Based Smart Photovoltaic Windows

To promote the development of ESBs, great efforts have been devoted to exploit SPWs with distinct compositions and structures by utilizing different kinds of smart materials ranging from organic to inorganic.^[^ ^25^, ^29^ ^]^ Herein, SPWs are categorized into two types according to their working principle. One type is solar cells containing stimuli‐responsive absorption component.^[^ ^31^, ^32^, ^33^ ^]^ The active absorption component enables SPWs photoelectric conversion characteristics. In addition, its stimuli‐responsive chromic behavior endows SPWs with the energy‐saving effect. To date, research work on stimuli‐responsive solar cells based SPWs is mainly focused on perovskites solar cells because of their several merits. The broad range of perovskite materials exhibit reversible color changing behaviors under external stimuli such as temperature,^[^ ^34^, ^35^ ^]^ light,^[^ ^36^, ^37^ ^]^ electrical field,^[^ ^38^, ^39^ ^]^ and stress,^[^ ^40^ ^]^ which affords an effective toolbox for SPWs design. Perovskites possess sharp absorption edge, low exciton binding energy, and modulable bandgap, which contribute to achieve high PCE.^[^ ^41^, ^42^ ^]^ Moreover, low material and production costs make perovskites solar cells bring enormous economic returns when they are applied for ESBs.^[^ ^43^ ^]^

**Figure 2:**
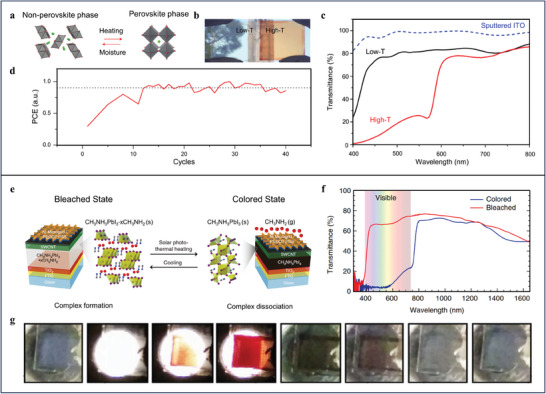
a) Schematic of the phase transition of inorganic halide perovskites by heating and exposure to moisture. Caesium and halide atoms are shown in green and red, respectively. b) Photograph of the low‐T phase (non‐colored) and high‐T (orange–red‐colored) thin films. c) The transmittance spectra of the semi‐transparent device in the low‐T (black curve) and high‐T (red curve) phases. d) The PCE of the high‐T phase over 40 transition cycles. a–d) Reproduced with Permission.^[^ ^31^ ^]^ Copyright 2018, Springer Nature. e) Schematic of the architecture of the SPWs and their switching process. f) Transmittance of the SPWs in bleached (red) and colored (blue) states as a function of wavelength. g) Optical images showing the transition of the SPWs from bleached to colored and back to bleached. e–g) Reproduced with Permission.^[^ ^32^ ^]^ Copyright 2017, Springer Nature.

Lin et al. demonstrates thermochromic SPWs featuring tunable transparency and electrical power generation by employing inorganic caesium lead iodide/bromide as absorption component.^[^ ^31^ ^]^ Thanks to thermal‐responsive phase transition behavior of inorganic caesium lead iodide/bromide (**Figure** **2**a), the SPWs exhibit high visible transparency (81.7%) at low temperature while low transparency (35.4%) at high temperature (Figure 2b,c). Attributing to the high thermal and environmental stability of the inorganic perovskite, the SPWs maintain more than 85% of the peak PCE (>7%) after up to 40 repeated cycles of transition (Figure 2d). Using methylammonium lead iodide–methylamine complex with low formation/dissociation energy as an absorption component, Wheeler et al. devises sunlight‐driven SPWs with relatively higher PCE (11.3%).^[^ ^32^ ^]^ Upon illumination, the photothermal effect of sunlight induces the dissociation of the methylammonium lead iodide‐methylamine complex, the SPWs display a colored state with an average visible light transmittance of 3%. Releasing the light source, the SPWs switch to a bleached state with an average visible light transmittance of 68% because the methylammonium lead iodide–methylamine complex is re‐formed at room temperature (Figure 2e–g). Caused by CH_3_NH_2_ loss during the process of the repeated formation/dissociation of the methylammonium lead iodide–methylamine complex, the PCE of the SPWs would gradually decrease. Although considerable work has been performed to improve the PCE and environmental stability of stimuli‐responsive solar cells based SPWs via material and structure optimization, there is an inevitable trade‐off between photovoltaic efficiency and light regulation performance.^[^ ^31^, ^32^, ^33^ ^]^

## 3 Chromic Unit and Passive Solar Cells Assembled Smart Photovoltaic Windows

The other type is integrated devices composed of chromic unit and solar cells.^[^ ^44^ ^]^ In this type SPWs, the chromic unit provides SPWs an energy‐saving effect attributed to its dynamically adjustable transparency under external stimuli. The solar cells are passive and solely act as an energy conversion component. Recently, the assembly of chromic units and passive solar cells has become the mainstream for designing advanced SPWs for several reasons. A variety of electrical‐,^[^ ^45^, ^46^ ^]^ thermal‐,^[^ ^47^, ^48^ ^]^ and photo‐responsive^[^ ^49^ ^]^ chromic materials can be utilized as chromic unit and seamlessly combined with various kinds of photovoltaic devices encompassing organic, dye‐sensitized, perovskite, and silicon solar cells. In addition, the trade‐off between photovoltaic efficiency and light regulation performance can be effectively overcome owing to the independent function of chromic units and solar cells.^[^ ^50^, ^51^ ^]^ By means of micro‐ and macro‐structure design, energy‐saving effect and photovoltaic efficiency can be optimized.^[^ ^52^, ^53^ ^]^ In this section, we review the state‐of‐the‐art technology for obtaining SPWs with autonomous and controllable operation mode, superior energy‐saving effect, high PCE, and long service life from the perspective of distinct stimuli‐responsive chromic materials and their combining form with solar cells.

### 3.1 Electrical‐Responsive Chromic Unit System

To date. electrical‐responsive chromic materials such as conducting polymers,^[^ ^54^, ^55^ ^]^ viologens,^[^ ^56^ ^]^ tungsten oxide (WO_3_),^[^ ^57^, ^58^, ^59^ ^]^ and metals^[^ ^60^ ^]^ have been widely used as the chromic unit to couple with solar cells for exploring SPWs due to their compelling traits. Their color changing behavior can be accurately, rapidly, and stably manipulated according to the needs of the occupant under complex environmental conditions.^[^ ^61^, ^62^ ^]^ The low direct current driven‐voltage makes them readily combine with solar cells for realizing the self‐powered feature required in high‐efficiency SPWs.^[^ ^63^ ^]^ Moreover, their electrical‐responsive nature enables them to be connected to the computer and internet equipment, holding the potential for constructing smart home systems in the future.^[^ ^64^ ^]^

#### 3.1.1 Conducting Polymers

**Figure 3:**
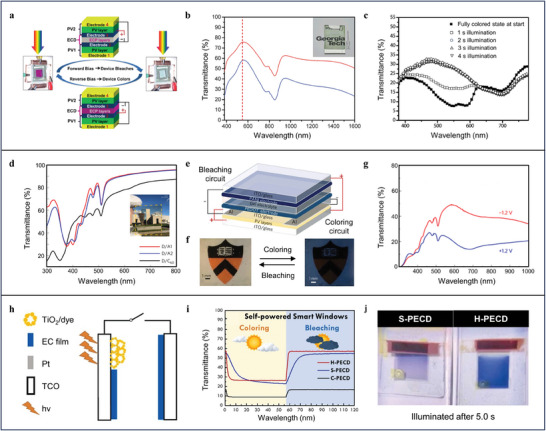
a) Photographs and schematic of the architecture of multi‐layer self‐powered SPWs at bleached and colored states. b) Transmittance spectra of a single organic solar cell (red) and two organic solar cells. Inset shows the photograph of the single organic solar cell. c) The transmittance spectra of the vertical SPWs illuminating for different times under AM 1.5 (1 sun). a–c) Reproduced with Permission.^[^ ^67^ ^]^ Copyright 2014, Wiley‐VCH. d) The transmittance spectra of active layer of organic solar cells which is composed of different electron acceptors. Inset shows the photograph of D/A1 active‐layer film on glass. e) Schematic of the stack geometry of the SPWs and circuit connections for powering the colored and bleached states. f) Photographs of the colored and bleached states of electrical chromic layer. g) Transmittance spectra of the SPWs at bleached (red) and colored (blue) states. d–g) Reproduced with Permission.^[^ ^68^ ^]^ Copyright 2017, Springer Nature. h) Schematic of the architecture of hybrid type SPWs. i) Dynamic transmittance responses of different type SPWs under illumination and dark. j) Photographs of separated and hybrid type SPWs under illumination for 5 s illustrating their distinct response speed. h–j) Reproduced with Permission.^[^ ^69^ ^]^ Copyright 2021, Elsevier.

Conducting polymers are functional polymeric materials possessing adjustable conductivity, low‐temperature processability, excellent mechanical properties.^[^ ^65^, ^66^ ^]^ The distinct optical performance of conducting polymers at oxidized and reduced states endows them with electrochromic behavior and, consequently, can be employed as a chromic unit of SPWs. Through sandwiching conducting polymer PProDOT‐(CH_2_OEtHx)_2_ as electrochromic layer between two separated organic solar cells, Dyer et al. demonstrates a vertical self‐powered SPW.^[^ ^67^ ^]^ The organic solar cells are able to provide forward and reverse bias to stimulate the bleaching and coloring of the electrochromic layer by connecting appropriate organic solar cells electrodes to electrochromic layer electrodes (**Figure** **3**a). The multiple solar cells connected in series certainly will greatly reduce the transmittance of SPW at bleached state (< 30%) and results in weak light adjusting capability (ΔT_max_ = 20%) that can hardly offer ideal energy‐saving effect (Figure 3b,c). To improve the light adjusting capability of SPWs for achieving superior energy‐saving performance, Navy et al. reports a scalable SPW based on an organic single‐junction solar cell and multi‐element conducting polymers composed of poly(2‐acrylamido‐2‐methyl‐1‐propane‐sulfonic acid) doped polyaniline and poly(3,4‐ethylenedioxythiophene):poly(styrenesulfonate).^[^ ^68^ ^]^ The single‐junction solar cell displays high transmittance in the visible region that is beneficial for giving SPWs the desired light adjusting capability (Figure 3d). In addition, it is capable of selectively harvesting near‐ultraviolet photons to produce high open‐circuit voltage (1.6 V) to power the dynamic chromic behavior of multi‐element conducting polymers (Figure 3e,f). Thanks to the high transmittance of the organic single‐junction solar cell and complementarity of multi‐element conducting polymers in electrochromic property, the developed SPWs exhibit desirable light adjusting capability (ΔT_max_ = 30%) (Figure 3g). Besides raising the transmittance of solar cells, the simplification of the structure of SPWs is an alternative to enhance their light adjusting capability. Chen et al. pioneers a hybrid type of self‐powered SPWs by utilizing PEDOT‐MeOH, a conducting polymer with electrocatalytic activity, as chromic unit and counter electrode of dye‐sensitized solar cells (Figure 3h).^[^ ^69^ ^]^ By this design, the SPWs of unique architecture show fast responsive behavior between colored and bleached states and moderate light adjusting capability (ΔT_max_ = 31.7%) (Figure 3i,j).

#### 3.1.2 Viologens

**Figure 4:**
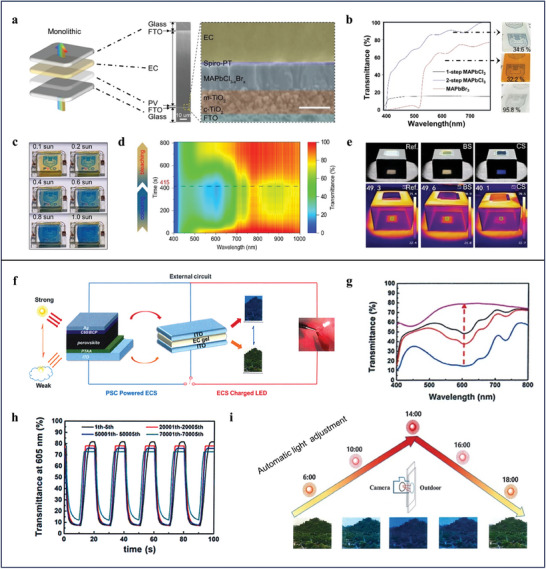
a) Schematic and cross‐section scanning electron microscope (SEM) images of two‐terminal monolithic integrated SPWs. b) Transmittance spectra and photographs of the perovskite films deriving from 1‐step MAPbCl_3_, MAPbBr_3_, and 2‐step MAPbCl_3_. c) Photographs of the developed SPWs at different sunlight intensities. d) Contour mapping diagram of real‐time full‐wavelength (400–1000 nm) transmission spectra under AM 1.5 G illumination. e) Profile and IR photographs of the SPWs at bleached and colored states, two FTO glass substrates used as reference. a–e) Reproduced with Permission.^[^ ^72^ ^]^ Copyright 2021, Springer Nature. f) Schematic diagrams of the architecture of the split type SPWs and their working principle. g) UV–vis spectra of all‐in‐one electrochromic devices powered by connected perovskite solar cells at different light intensities. h) Cycling stability of the all‐in‐one electrochromic devices switching between colored and bleached states. i) Automatic light adjustment of self‐powered SPWs in real‐time. f–i) Reproduced with Permission.^[^ ^73^ ^]^ Copyright 2021, Springer Nature.

Viologens are organic small molecule electrochromic materials having good optical contrast, high coloring efficiency, and excellent oxidation‐reduction stability which offers a promising platform for developing SPWs.^[^ ^70^, ^71^ ^]^ In order to realize integrated simple architecture, scalable producibility, and exceptional energy‐saving effect, Liu presents a vertical tandem structured self‐powered SPWs by combining full‐transparent perovskite solar cells and viologen gels (**Figure** **4**a).^[^ ^72^ ^]^ High transparency of the perovskite solar cells is attributed to perovskite layer with pristine transmittance up to 76% fabricated by a halide‐exchanging technology (Figure 4b). Under sunlight irradiation, the full‐frame SPWs can generate voltage to charge the viologen gels to obtain colored state (Figure 4c). Notably, the SPWs can switch from highly transparent state (85%, 600 nm) to colored state with transmittance of 20% (600 nm) under 1.0 sun illumination for 300 s. Moreover, high contrast ratio (> 30%) on average visible‐light transmittance between colored and bleached states gives the SPWs prime energy‐saving performance (Figure 4d,e). However, long coloring and bleaching time (>300 s) and low PCE (0.63%) restrict their real‐world application. Instead of assembling viologen gels into highly transparent perovskite solar cells to devise monolithic SPWs, Ling et al. develops a split type SPWs by directly linking viologen based all‐in‐one electrochromic device to semi‐transparent perovskite solar cells (Figure 4f).^[^ ^73^ ^]^ Compared to SPWs with integral structure, chromic unit and solar cells are relatively independent and having more freedom to take the role of energy‐saving and energy conversion in the split type SPWs. For instance, opaque perovskite solar cells with high PCE (> 17.3%) can be selected as functional component of SPWs to produce high electrical output for triggering electrochromic behavior of the viologens and supplying daily electricity consumption. Via introducing alkynyl group into the viologens, viologen gels, acting as the chromic unit, can rapidly and stably switch between colored and bleached states (Figure 4g,h). The coloring and bleaching time are ≈2.5 s. After 7000 cycles, high contrast ratio (ΔT_max_ = 60.9%) between colored and bleached states remains which ensures the SPWs extraordinary energy‐saving performance. Last but not least, the SPWs have self‐adaptable capability because the color changing behavior of the viologen gels is determined by photocurrent closely related to the intensity of the sunlight (Figure 4i).

#### 3.1.3 WO_3_

WO_3_, a typical transition metal oxide, are the first electrochromic materials that are utilized to couple with solar cells to explore SPWs because of several merits. They are rich in natural resources and cheap on earth.^[^ ^74^ ^]^ They have high coloring efficiency, fast response, good cycle stability.^[^ ^75^ ^]^ Distinct from organic electrochromic materials, they have light modulation ability both in visible and near‐infrared (NIR) regions.^[^ ^76^, ^77^ ^]^ As visible and NIR light take account ≈52.4% and 43.0% of the solar radiation energy respectively, WO_3_ acting as the chromic unit with broadband modulation feature hold great potential to enable SPWs prominent energy‐saving effect.^[^ ^78^ ^]^

**Figure 5:**
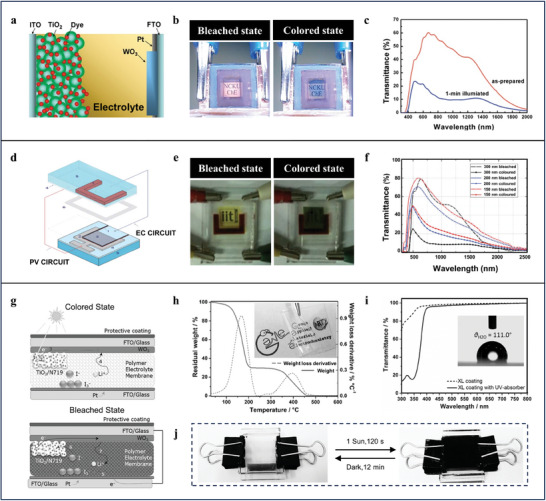
a) Schematic of the architecture of the SPWs applying patterned WO_3_/Pt as the counter‐electrode. b) Photographs of the SPWs in the as‐prepared (left) and colored (right) states. c) Transmittance spectra of the SPWs in the as‐prepared and colored state. a–c) Reproduced with Permission.^[^ ^79^ ^]^ Copyright 2009, American Chemical Society. d) Schematic of the architecture of the SPWs with two available circuits. e) Photographs of the SPWs in the bleached (left) and colored (right) states. f) Transmittance spectra of the SPWs with different thickness of the WO_3_ in the as‐prepared and colored states. d–f) Reproduced with Permission.^[^ ^80^ ^]^ Copyright 2011, The Royal Society of Chemistry. g) Sketched architecture of the proposed quasi‐solid SPWs and their working principle. h) Thermogravimetric analysis (TGA) thermogram of polymeric electrolyte. Inset shows the photograph of polymeric electrolyte after 50 cycles bending. i) Transmittance spectra of the protective coating. Inset shows static water contact angle of the protective coating. j) Photographs of the quasi‐solid SPWs in the bleached (left) and colored (right) states. g–j) Reproduced with Permission.^[^ ^81^ ^]^ Copyright 2016, Wiley‐VCH.

By applying patterned WO_3_/Pt as the counter‐electrode of dye‐sensitized solar cells, Wu et al. presents a fast‐switching self‐powered SPW (**Figure** **5**a).^[^ ^79^ ^]^ Replacing commonly used Pt electrode with this electrochromic electrode, the SPWs possess photovoltaic characteristics comparable to those of dye‐sensitized solar cells. Assisting by the catalytic action of the Pt, the SPWs can switch to colored state under the light illumination at short circuit and back to bleached state at open circuit both in 4 s (Figure 5b). Via employing WO_3_ as the chromic unit, broadband modulation performance is achieved in the SPWs (ΔT_max_ = 40.0%) (Figure 5c). For practical application, the light adjusting and electric power generation functionalities of the SPWs should be independent and complementary for achieving maximized energy‐saving effect. In the above architecture design, the coloring and electric power generation of the SPWs is usually simultaneous. It means that the SPWs always keep at the colored state and cannot freely switch between colored and bleached states according to the change of surrounding environments if they perform the electric power generation. To address this issue, Cannavale et al. explores special SPWs with two available circuits that separately realize light adjusting and electric power generation behaviors (Figure 5d).^[^ ^80^ ^]^ These two circuits are generated by devising a C‐shaped counter electrode which bounds and electrically separates to a square region occupied by WO_3_ film. By optimizing the composition of the electrolyte and thickness of WO_3_ film, the contrast ratio of ΔT_max_ = 60.0% and PCE of 6.55% are achieved in designed SPWs (Figure 5e,f). As commonly used electrolytes in dye‐sensitized solar cells is liquid, the volatility, photoinstability, corrosion, and leakage of liquid electrolytes should be taken into consideration when utilizing dye‐sensitized solar cells as photovoltaic component to design SPWs. To this end, Bella et al. demonstrates a new paradigm for designing thermally, electrochemically, and photochemically stable SPWs (Figure 5g).^[^ ^81^ ^]^ In this system, a transparent, flexible, and thermally stable quasi‐solid polymeric film containing lithium and iodide ions is applied as the electrolyte to eliminate the volatility, corrosion, and leakage issues often occurring in liquid electrolytes (Figure 5h). Furthermore, an ultraviolet absorption, visible transparent, and hydrophobic fluoropolymeric thin film with a water contact angle of 111.0° is utilized as an external protective coating in the SPWs for enhancing the photostability and easy‐cleaning (Figure 5i). Although the utilization of the quasi‐solid polymeric electrolyte can improve the safety, stability and long‐term operational life of the SPWs, the response speed and PCE (0.42%) are greatly reduced at the same time (Figure 5j).

**Figure 6:**
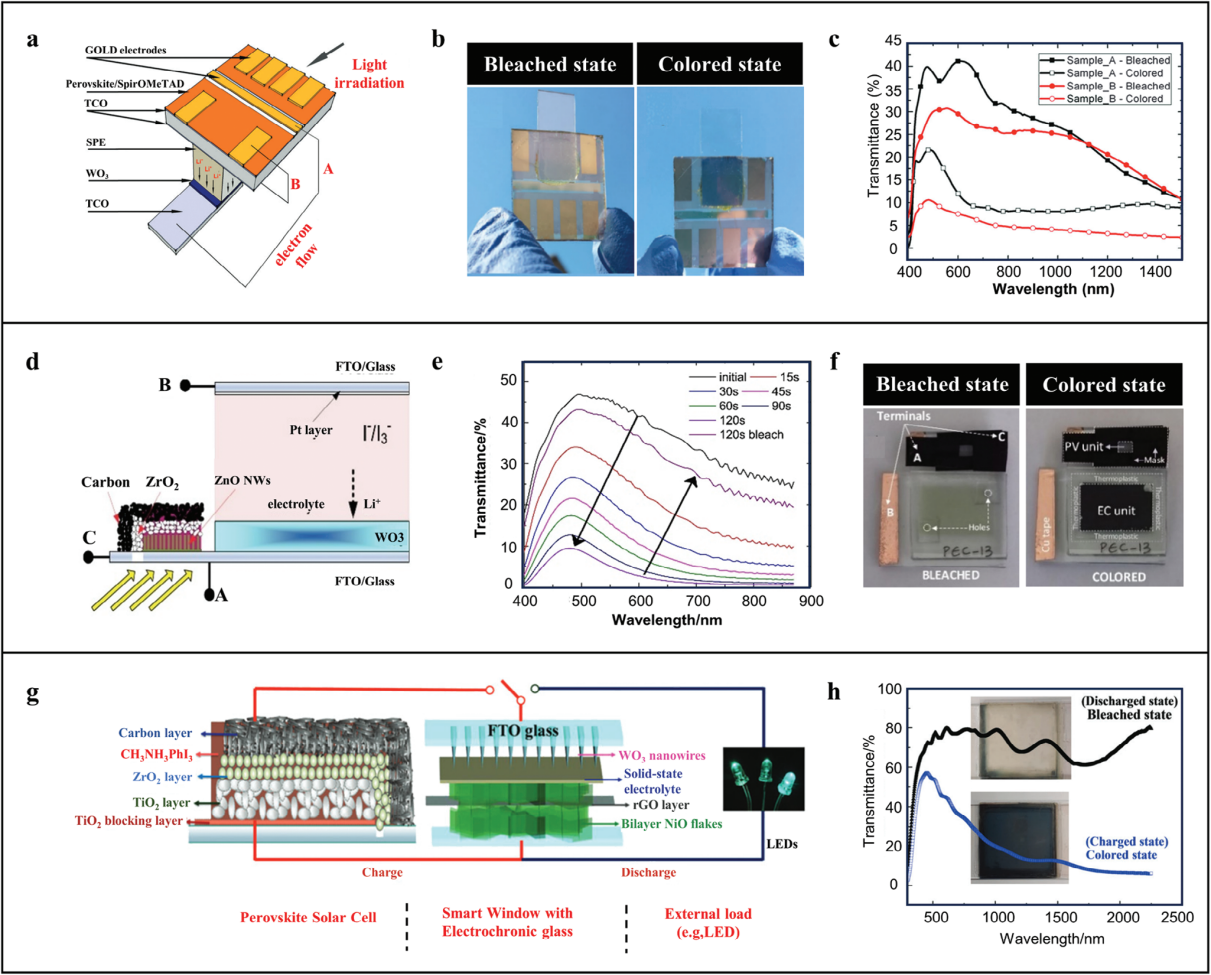
a) Schematic of the architecture of the tandem structured self‐powered SPWs and their working principle. b) Photographs of the SPWs in the bleached (left) and colored (right) states. c) Transmittance spectra of the SPWs with different perovskite microstructured in the bleached and colored states. a–c) Reproduced with Permission.^[^ ^82^ ^]^ Copyright 2015, Royal Society of Chemistry. d) Schematic of the architecture of the “partly covered” SPWs. e) Transmittance spectra of the SPWs during coloration under 1000 w m^−2^ (AM 1.5G) and bleaching under short circuit. f) Photographs of the SPWs in the bleached (left) and colored (right) states. d–f) Reproduced with Permission.^[^ ^83^ ^]^ Copyright 2020, Elsevier. g) Schematic of the architecture of the proposed SPWs and their working principle. h) Transmittance spectra and photographs of the electrochromic batteries in the bleached and colored states. g,h) Reproduced with Permission.^[^ ^84^ ^]^ Copyright 2016, Royal Society of Chemistry.

All solid perovskite solar cells make it possible to solve this trade‐off. Cannavale et al., the first time, employs perovskite solar cells as photovoltaic components to combine with WO_3_ for exploiting tandem structured self‐powered SPWs (**Figure** **6**a,b).^[^ ^82^ ^]^ Being beneficial from all‐solid integral structure, the SPWs possess extraordinary safety and stability besides relatively faster response speed and higher PCE (5.5%). However, it is difficult to make a balance between the light adjusting performance and PCE in this tandem structured SPWs (Figure 6c). Moreover, the perovskite solar cells are restricted to transparent and semitransparent ones, which are unfavorable to the pluralistic design of the SPWs. Syrrokostas et al. proposes a “partly covered” SPW by adopting opaque carbon‐based perovskite solar cells and WO_3_ as the photovoltaic component and chromic unit, respectively (Figure 6d).^[^ ^83^ ^]^ Due to this unique architecture, the intrinsic light adjusting capability of the chromic unit, WO_3_, is not influenced by the transmittance of the photovoltaic component and is completely retained in the SPWs (Figure 6e). As a result, transparent, semitransparent, and opaque perovskite solar cells can all function as photovoltaic component for design SPWs for a particular requirement. In addition, the photovoltaic component taking ultra‐small area (4%) of the whole SPWs can produce open circuit voltage to drive optical modulation behavior of the WO_3_ (Figure 6f). Although the PCE (2.78%) of the SPWs is relatively lower, the cost of carbon electrodes applied in opaque perovskite solar cells is much cheaper than that of precious metal transparent electrodes used in transparent or semitransparent ones, which can remarkably reduce the manufacturing cost of the SPWs and promote their real‐world application. To make the SPWs more efficient, Zhou et al. devises a multifunctional SPWs based on perovskite solar cells and electrochromic batteries (Figure 6g).^[^ ^84^ ^]^ In this multifunctional SPWs, the electrical energy generated by the perovskite solar cells can excite the color changing behavior of the electrochromic batteries for giving energy‐saving effect and also be stored in the electrochromic batteries for reutilization. More importantly, WO_3_ and NiO of special nanostructures not only act as electrodes to afford excellent electrochemical performance but also as electrochromic materials to offer distinguished light adjusting capability, especially in the NIR region (Figure 6h).

### 3.2 Thermal‐Responsive Chromic Unit System

#### 3.2.1 Vanadium Oxide (VO_2_)

**Figure 7:**
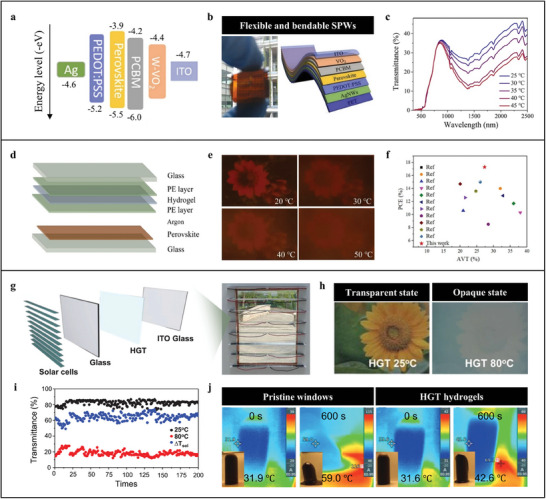
a) Energy level diagram of the SPWs. b) Schematic of the architecture (right) and photograph (left) of the flexible and bendable SPWs. c) Transmittance spectra of the SPWs at different temperatures indicating their NIR modulation behavior. a–c) Reproduced with Permission.^[^ ^47^ ^]^ Copyright 2022, Elsevier. d) Schematic of the architecture of the proposed SPWs. e) Photographs of the SPWs at different temperatures. f) Comparative PCE‐AVT charts for the devised SPWs (red star). d–f) Reproduced with Permission.^[^ ^48^ ^]^ Copyright 2022, Elsevier. g) Schematic of the architecture (left) and photograph (gight) of the multi‐layer louver structured SPWs. h) Optical photos of the HGT hydrogels at 25 and 80 °C. i) Cycling testing for the HGT hydrogels. j) Thermal infrared images and optical photos of the ordinary window‐based and HGT hydrogel‐based house under the simulated sunlight at 0 and 600 s. g–j) Reproduced with Permission.^[^ ^89^ ^]^ Copyright 2022, Wiley‐VCH.

With passive and zero‐energy input characteristics, thermal‐responsive chromic materials have been considered promising chromic units for designing SPWs. Due to the metal–insulator transition, VO_2_ is a typical thermal‐responsive material with NIR modulation capability.^[^ ^85^, ^86^ ^]^ Meng et al. demonstrates an integrated flexible SPW by combing organic perovskite solar cells and tungsten doped VO_2_ (**Figure** **7**a).^[^ ^47^ ^]^ The doping of tungsten decreases the conduction band of VO_2_ to −4.4 eV so that it can be utilized as a suitable buffer layer to solve energy level mismatch between the electron transport layer and the work function electrode for achieving high PCE (16.1%) (Figure 7b). Furthermore, tungsten doping also lowers the metal–insulator transition temperature of VO_2_ to 40 °C, consequently giving the SPWs favorable NIR modulation of 10.7% near room temperature (Figure 7c). However, comparatively lower transparency and deficiency of light adjusting capability of VO_2_ in the visible region is a bottleneck for applying it as the chromic unit to develop SPWs with desirable energy‐saving effect.

#### 3.2.2 Hydrogels

Hydrogels are classic organic thermal‐responsive chromic materials with outstanding solar modulation ability, cycle stability, tunable lower critical solution temperature (LCST), and low toxicity.^[^ ^6^, ^87^, ^88^ ^]^ Meng et al. presents a tandem SPW by coupling hydroxypropyl cellulose (HPC) hydrogels and semitransparent perovskite solar cells (Figure 7d).^[^ ^48^ ^]^ Thanks to near room temperature LCST of HPC hydrogels, the SPWs can transform from semitransparent sate to opaque state over the temperature of 40 °C with the solar energy modulation (T_sol_) of 15.7% (Figure 7e). By assembling with semitransparent perovskite solar cells, high PCE of 17.5% is achieved the SPWs (Figure 7f). However, the lower transparence of the semitransparent perovskite solar cells inevitably sacrifices the initial transmittance and T_sol_ of the SPWs. For this, Niu reports a multifunctional SPW integrating energy‐saving, active control, and anti‐freezing via combing louver structured silicon solar cells, host‐guest thermal‐responsive hydrogels (HGT), and indium oxides (ITO) glass (Figure 7g).^[^ ^89^ ^]^ Beneficial from this unique design, highly transparent initial state (ΔT_lum_, 88.68%) and ultrahigh T_sol_ of 54.02% of the chromic unit HGT hydrogels are well inherited by the SPWs which affords them excellent energy‐saving performance (Figure 7h–j). The silicon solar cells, as the photovoltaic component, endow the SPWs with stably high PCE (18.24%). In addition, active control and anti‐freezing are realized in the SPWs due to the high electrical output of the silicon solar cells and electro–thermal effect of ITO.

#### 3.2.3 Liquid Crystals (LCs)

**Figure 8:**
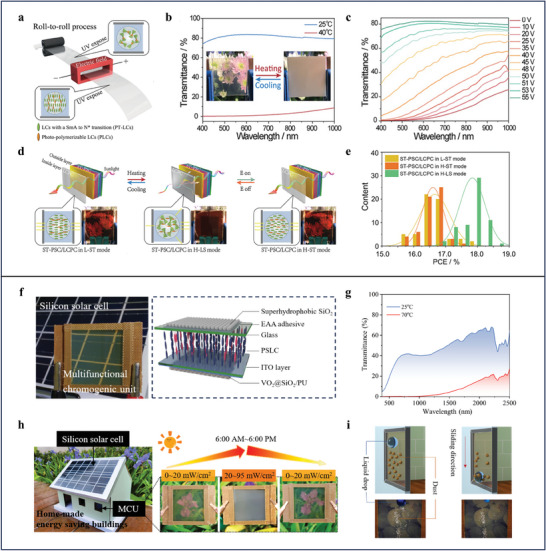
a) Schematic illustration of roll‐to‐roll process for preparing multi‐responsive LC composite films. b) UV–vis transmittance spectra of LC composite films at SmA (25 °C) and N^*^ phase (40 °C). Inset shows the photographs of LC composite films at at these two phases. c) UV–vis spectra of N^*^ phase LC composite films under various voltages. d) Schematic illustration of the principle for the SPWs working at three different modes. e) PCE distribution of the SPWs in three different working modes. a–e) Reproduced with Permission.^[^ ^90^ ^]^ Copyright 2019, Wiley‐VCH. f) Photograph of the split‐type SPWs and schematic of the architecture of the multifunctional chromic unit. g) UV–vis transmittance spectra of the the multifunctional chromic unit at the temperature of 25 and 70 °C. h) Photographs demonstrating the self‐driven behavior of the SPWs under sunlight irradiation. i) Diagrammatic sketches and photographs that represent the self‐cleaning behavior of the SPWs. f–i) Reproduced with Permission.^[^ ^93^ ^]^ Copyright 2024, Wiley‐VCH.

Despite great efforts have been devoted to employ thermal‐responsive materials such as VO_2_ and hydrogels as chromic units for exploring high efficiency SWPs, these kinds SPWs cannot freely manipulate their transparency according to the demand of the occupant.^[^ ^47^, ^48^ ^]^ For example, the SPWs will turn opaque when ambient temperature is over the critical transition temperature of VO_2_ or hydrogels. At this relatively higher temperature, the SPWs will keep an opaque state and cannot return to the initial transparent state, which is needed for the occupant to enjoy the outdoor scenery. To solve this issue, our group demonstrates a high‐efficiency and reliable SPWs with multiple working modes enabled by multi‐responsive LC composite films and semi‐transparent solar cells.^[^ ^90^ ^]^ The LC composite films can be large‐area mass produced by roll‐to‐roll process (**Figure** **8**a). They exhibit thermal‐responsive and electrical‐responsive chromic behaviors because they are a coexistent system containing polymer‐dispersed LC and polymer stabilized LC.^[^ ^91^, ^92^ ^]^ When the ambient temperature is over 40 °C, the LC composite films, functioning as the chromic unit, switch from a transparent state (79% visible transparency) to an opaque state (3% visible transparency) due to the temperature‐induced SmA to N^*^ phase transition for offering energy‐saving effect (Figure 8b). Notably, the LC composite films can recover to the initial transparent state under external voltage at comparably higher ambient temperature (> 40 °C), which is hardly realized in other thermal‐responsive chromic materials (Figure 8c). Thanks to the multi‐responsive characteristic of the LC composite films, the devised SPWs obtain multiple working modes affording flexible light adjusting capability required for the daily life of the occupant (Figure 8d). Last but not least, the PCE of the SPWs can be greatly enhanced in H‐LS mode because the strong light‐scattering effect of the LC composite films at an opaque state effectively increases the optical path length of the light and solar energy harvesting of the semi‐transparent solar cells (Figure 8e). Although the utilization of the multi‐responsive LC composite films as chromic units can give the SPWs maneuverable light adjusting ability, additional electric energy consumption is needed. More importantly, the lack of NIR adjusting capability for the multi‐responsive LC composite films limits the energy‐saving effect of SPWs. To maintain the flexible light adjusting capability meanwhile, make SPWs more energy‐efficient, our group presents a split‐type SPWs with broadband modulation, self‐driven, and self‐cleaning behaviors by the combination of a silicon solar cells and a multifunctional chromic unit (Figure 8f).^[^ ^93^ ^]^ The multifunctional chromic unit is mainly composed of polymer stabilized LC, VO_2_@SiO_2_ nanoparticle, ITO, and SiO_2_ coating which are visible light adjusting layer, NIR light adjusting layer, electrical heating layer, and superhydrophobic layer respectively. The synergy of thermal‐responsive polymer stabilized LC and VO_2_@SiO_2_ nanoparticle offers a broadband modulation feature, which is needed to achieve ideal energy‐saving effect in SPWs (Figure 8g).^[^ ^94^ ^]^ Owing to the pre‐designed phase transition temperature (60 °C) of polymer‐stabilized LC and VO_2_@SiO_2_ nanoparticle, distinguished electrothermal property of the ITO, and high open‐circuit voltage of the silicon solar cells, the broadband modulation behavior of the SPWs can be stimulated by the electrical energy produced by the silicon solar cells via harvesting solar energy (Figure 8h). In addition, the self‐cleaning behavior of the SPWs endowed by the superhydrophobic SiO_2_ coating can effectively reduce the water resource consumption for cleaning, meanwhile ensuring optimal visibility on rainy days (Figure 8i).

### 3.3 Other‐Responsive Chromic Unit System

Besides electrical‐ and thermal‐responsive chromic materials, photo‐responsive chiral nematic LCs and gas‐responsive WO_3_ have been attempted to be applied as chromic units for exploiting SPWs.^[^ ^49^, ^95^ ^]^ For example, Kwon et al. devises a photo‐responsive chiral nematic LC with switchable optical properties and tries to integrate it with dye‐sensitized solar cells for developing SPWs.^[^ ^49^ ^]^ Although high PCE (7.97%) is gained by modulating the macrostructure of the SPWs, they can hardly fulfill the energy‐saving effect because they are initially opaque and transparent state has to be gained by the light illumination. Yao et al. proposes a fast response and high‐efficiency SPW by adopting gas‐responsive WO_3_/Pt as a back reflector of semitransparent polymer solar cells.^[^ ^95^ ^]^ The WO_3_/Pt back reflector layer exhibits colored and bleached states under hydrogen and oxygen atmosphere respectively indicating it can function as a gas‐responsive chromic unit giving the SPWs energy‐saving effect. However, the use of the hydrogen requires severe external equipment and potentially brings safety hazard.

## 4 Concluding Remarks

SPWs have emerged as promising candidates for exploring ESBs because they can offer energy‐saving effect and produce electrical power output by managing and harnessing solar energy. To date, a series of SPWs with distinct working principles, functional components, architectures, energy‐saving and photovoltaic performance have been designed and summarized in **Table** **1** and **Table** **2**. To propel the real‐world application of SPWs in high efficiency ESBs, the following points concerning about future studies on SPWs are given.

**Table 1:** Summary of stimuli‐responsive solar cells based smart photovoltaic windows.

Ref.	Modulation mechanisms	SPW structure	AVT_b_ ^a)^ [%]	AVT_c_ ^b)^ [%]	ΔAVT ^c)^ [%]	PCE_b_ ^d)^ [%]	PCE_c_ ^e)^ [%]	Stability^f)^
[31]	Thermochromic	Tandem	81.7	35.4	46.3	0.1	4.7	10
[32]	Thermochromic	Tandem	68.0	>3.0	65.0	0.0	11.3	20 (I_sc_ reduced to 18%)

^a)^ AVT_b_: Average visible transmittance at bleached state;

^b)^ AVT_c_: Average visible transmittance at colored state;

^c)^ ΔAVT: Visible light modulation ability;

^d)^ PCE_b_: Power conversion efficiency at bleached state;

^e)^ PCE_c_: Power conversion efficiency at colored state;

^f)^ Stability: The number of cycles after which the photovoltaic properties can still be maintained.

**Table 2:** Summary of chromic unit and passive solar cells assembled smart photovoltaic windows.

References	Chromic unit	PV devices	SPW structure	T_b_ ^a)^ [%]	T_c_ ^b)^ [%]	AVT_b_ ^c)^ [%]	AVT_c_ ^d)^ [%]	ΔT ^e)^ [%]	t_c_ ^f)^ [s]	t_b_ ^g)^ [s]	Peak PCE [%]	Stability^h)^
[67]	PProDOT‐(CH_2_OEtHx)_2_	S‐T^i)^ Organic SC	Tandem	≈28.4 @550 nm	≈7.9 @550 nm	–	–	≈20.5 @550 nm	3.0	2.0	0.5	–
[68]	PEDOT:PSS+ PANI‐PAAMPSA	Organic SC	Tandem	≈44.4 @650 nm	≈14.7 @650 nm	–	–	≈29.7 @650 nm	40.0	40.0	1.5	–
[69]	PEDOT‐MeOH	DSC	Tandem	57.4 @600 nm	25.8 @600 nm			31.7 @600 nm	5.5	3.3	2.2	–
[72]	HV(TF‐SI)_2_	F‐T^j)^ Perovskite SC	Tandem	≈83.2 @600 nm	≈18.2 @600 nm	–	–	≈65.0 @600 nm	300.0	200.0	0.6	10 100 (ΔT reduced by ≈34%)
[73]	DPV	Perovskite SC	Split	≈79.0 @605 nm	≈14.1 @605 nm	–	–	≈64.9 @605 nm	2.7	27.6	18.3	70 000 (ΔT reduced by ≈19%)
[79]	WO_3_	DSC	Tandem	≈55.8 @788 nm	≈11.5 @788 nm	–	–	≈44.3 @788 nm	4.0	60.0	0.5	200
[80]	WO_3_	DSC	Tandem	≈65.5 @780 nm	≈9.2 @780 nm	–	–	≈56.3 @780 nm	5.0	10.0	6.6	10
[81]	WO_3_	DSC	Tandem	≈66.4 @600 nm	≈3.5 @600 nm	–	–	≈62.9 @600 nm	120.0	720.0	0.4	–
[82]	WO_3_	S‐T Perovskite SC	Tandem	≈29.5 @600 nm	≈7.5 @600 nm	16.0	5.5	≈22.0 @600 nm	15.0–20.0	15.0–20.0	5.5	–
[83]	WO_3_	Perovskite SC	Tandem	44.0 @T_lum_	5.0 @T_lum_	–	–	39.0 @T_lum_	120.0	120.0	2.8	–
[84]	WO_3_	Perovskite SC	Split	≈77.2 @750 nm	≈34.2 @750 nm	–	–	≈43.0 @750 nm	2.5	2.6	11.9	–
[47]	VO_2_	S‐T Perovskite SC	Tandem	≈29.5 @1500 nm	≈13.0 @1500 nm	25.5	25.5	≈16.5 @1500 nm	–	–	16.1	–
[48]	HPC Hydrogel	S‐T Perovskite SC	Tandem	≈67.5 @750 nm	≈35.4 @750 nm	27.3	10.4	≈32.1 @750 nm	–	–	17.5	
[89]	HPC Hydrogel	c‐Si SC	Split	≈89.6 @750 nm	≈5.8 @750 nm	–	–	≈83.8 @ 750 nm	–	–	18.2	200
[90]	LC	S‐T Perovskite SC	Tandem	≈15.0 @700 nm	≈1.2 @700 nm	79.0	3.0	≈13.8 @700 nm	–	–	18.0	50
[93]	LC+VO_2_	c‐Si SC	Split	47.0 @700 nm	0.5 @700 nm	45.4	6.9	46.5 @700 nm	–	–	23.0	500
[49]	LC	DSC	Tandem	–	–	–	–	–	60	–	8.0	–
[95]	WO_3_	S‐T Polymer SC	Tandem	≈87.2 @600 nm	≈51.6 @600 nm	33.8	25.4	≈35.6 @600 nm	0.92	0.85	10.2	15

^a)^ T_b_: Light transmittance at bleaching;

^b)^ T_c_: Light transmittance at coloring;

^c)^ AVT_b_: Average visible transmittance at bleaching;

^d)^ AVT_c_: Average visible transmittance at coloring;

^e)^ ΔT: Light modulation ability;

^f)^ t_b_: Time of bleaching;

^g)^ t_c_: Time of coloring;

^h)^ Stability: The number of cycles after which the light modulation properties can still be maintained;

^i)^ S‐T: Semi‐Transparent;

^j)^ F‐T: Full‐Transparent.

Recently, the development of stimuli‐responsive solar cells has been considered as an effective strategy for devising SPWs (Table 1). However, the key issue is the trade‐off between photovoltaic efficiency and light regulation performance caused by the phase transition of the stimuli‐responsive absorption component. This crucial issue can be overcome in tandem solar cells containing stimuli‐responsive and passive absorption components. The passive absorption component enables stable photovoltaic efficiency whether the SPWs are in colored or bleached states.

In addition, assembling various electrical‐, thermal‐, photo‐, and gas‐responsive chromic materials and passive solar cells is a fantastic approach for exploiting SPWs (Table 2). In this system, electrical‐responsive chromic materials such as conducting polymers, viologens, and WO_3_ are easily coupled with solar cells because of their actuating mode. The optical modulation behavior of the electrical‐responsive chromic materials can be accurately regulated by the electric energy output of the solar cells. However, complicated structure including transparent electrodes, ion transport layer, and ion storage layer are usually required for electrical‐responsive chromic materials to build up SPWs. To simplify the macrostructure, researchers attempt to place the electrical‐responsive chromic materials on the electrodes of dye‐sensitized solar cells due to their similar architecture. Furthermore, the solid electrolytes are utilized to solve unsafety and instability of the SPWs brought by the volatility, corrosion, and leakage of liquid electrolytes. Beyond providing the SPWs superb safety and stability, great efforts are still needed to develop novel solid electrolytes to afford the SPWs fast responsive speed and exceptional photovoltaic performance. Besides electrical‐responsive chromic materials, thermal‐responsive chromic materials such as VO_2_, hydrogels, and LCs have been widely used as the chromic unit to combine with solar cells for devising SPWs because they can automatically modulate solar energy according to the change of ambient temperature for giving the SPWs energy‐saving effect without any additional energy input. In this type of SPWs, flexible optical modulation capability that the transparency of the SPWs can be manipulated based on the occupants’ need is crucial for their practical application. For this, our group design multi‐responsive LC composite film and multi‐functional chromic unit with suitable phase transition temperature. Although few works have been tried to developing SPWs based on photo‐and gas‐responsive chromic materials, their energy‐saving performance far from electrical‐ and thermal‐responsive ones or requires severe external equipment.

Despite exciting progress in this cutting‐edge research field, it is still in the initial stage. Overall, a cost‐effective, stable, and large‐area preparable smart material system is urgently to be developed for the practical application of SPWs. The thermal radiation of solar energy is broadband, mainly composed of solar heating in the visible (380–780 nm), NIR (780–2500 nm) wavelength regions, and the spontaneous emission in the mid‐infrared (MIR, 8–13 µm) wavelength region.^[^ ^96^ ^]^ For achieving ideal energy‐saving effect, broadband modulation performance of SPWs crossing visible and NIR regions still needs the optimization. Moreover, transparent radiation cooling materials and structures that can regulate thermal radiation of solar energy within MIR range have not yet been reported in SPWs but should be considered in the future work. Lastly, multifunctional integration, for example, enabling SPWs privacy protection, energy storage, and self‐cleaning features are the trend and direction for the development of SPWs.

## Conflict of Interest

The authors declare no conflict of interest.
